# Biomechanical Effects of Aspect Ratio of the Knee during Outside-In Anterior Cruciate Ligament Reconstruction Surgery

**DOI:** 10.1155/2021/3454475

**Published:** 2021-09-04

**Authors:** Tae Soo Bae, Byeong Chan Cho, Dai-Soon Kwak

**Affiliations:** ^1^Department of Biomedical Engineering, Jungwon University, 85, Munmu-ro, Goesan-eup, Goesan-gun, Chungcheongbuk-do 28024, Republic of Korea; ^2^Catholic Institute for Applied Anatomy, Department of Anatomy, College of Medicine, The Catholic University of Korea, 222, Banpo-daero, Seocho-gu, Seoul 06591, Republic of Korea

## Abstract

We analyzed tunnel length, graft bending angle, and stress of the graft according to tunnel entry position and aspect ratio (ASR: ratio of anteroposterior depth to mediolateral width) of the articular surface for the distal femur during single-bundle outside-in anterior cruciate ligament reconstruction (ACLR) surgery. We performed multiflexible body dynamic analyses with four ASR (98, 105, 111, and 117%) knee models. The various ASRs were associated with approximately 1 mm changes in tunnel length. The graft bending angle increased when the entry point was far from the lateral epicondyle and was larger when the ASR was smaller. The graft was at maximum stress, 117% ASR, when the tunnel entry point was near the lateral epicondyle. The maximum stress value at a 5 mm distance from the lateral epicondyle was 3.5 times higher than the 15 mm entry position, and the cases set to 111% and 105% ASR showed 1.9 times higher stress values when at a 5 mm distance compared with a 15 mm distance. In the case set at 98% ASR, the low-stress value showed a without-distance difference from the lateral epicondyle. Our results suggest that there is no relationship between the ASR and femoral tunnel length. A smaller ASR causes a higher graft bending angle, and a larger ASR causes greater stress in the graft.

## 1. Introduction

The anterior cruciate ligament (ACL) in the knee joint is a frequently damaged ligament. An injured ACL has been reported to not only cause functional instability of the knee joint but also cause damage to the articular cartilage and secondary injuries to the peripheral tissue of the knee joint [1, 2]. Anterior cruciate ligament reconstruction (ACLR) is often necessary to restore an injured ACL and to restore patients to their normal activities. To date, clinical studies have not shown that ACLR reliably prevents cartilage lesions and restores knee stability to normal values [3, 4]. The femoral tunnel position should be considered as an important parameter when planning ACLR to restore knee stability [5, 6].

Various techniques for creating a femoral tunnel for ACLR have been proposed and used, but the outside-in technique is used widely due to its various advantages [7]. The outside-in tunnelling technique uses a specially designed indicator to determine direction and entry point. The instrument indicates the ACL footprint and the tunnel entry point at the lateral epicondyle. The surgeon creates a tunnel by drilling in the direction guided by the instrument, and the operator can adjust the tunnel entry position at the lateral epicondyle. Therefore, many studies have been conducted on the effect of tunnel position on surgical outcome, and the femoral tunnel position is an important parameter when planning ACLR to restore knee stability [8]. Also, the femoral tunnel position has been reported to influence biomechanical characteristics of an implanted graft, and it affects knee function during postoperative rehabilitation [9]. Studies have been conducted to determine the optimal femoral tunnel position during ACL surgery. Shino et al. attempted to determine the anatomical femoral tunnel position of an implanted graft using arthroscopy without bone incision in patients with chronic ACL insufficiency [10]. Zauleck et al. analyzed the ACL footprint site based on the shape of the lateral intercondylar ridge and the lateral bifurcate ridge [11]. Nam-Ki and Jong-Min compared ACLR surgical methods using the transtibial, anteromedial portal, and outside-in techniques to determine the optimal femoral tunnel position [12]. However, these studies had limitations. They only performed static analyses or simulations at certain positions.

**Figure 1:**
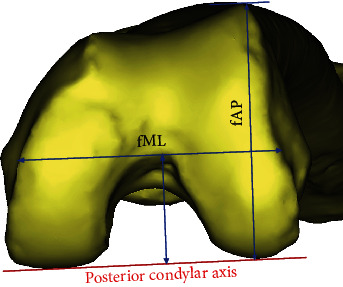
Measurement parameters for calculating the ASR. fAP indicates the height of the lateral condyle, and fML is the mediolateral width at the intercondylar notch parallel to the posterior condylar axis. ASR is defined as fML/fAP × 100.

To address this issue, Kang and Bae conducted a study to determine the femoral tunnel position under continuous knee movement during ACLR surgery, although only one knee model was used [6]. In contrast, there are numerous studies of knee morphology in anatomy and orthopaedics [13, 14]. Many morphological studies have been conducted to design an optimal artificial joint shape. Sex differences, population differences, and size mismatch were mainly studied, and the studies addressed the issue of anatomical characteristics, aspect ratio (ASR: ratio of anteroposterior depth to mediolateral width, as shown in Figure 1), and differences among the knee [15, 16]. However, most of the biomechanical studies were conducted without considering the knee ASR. The objective of this study was to analyze the biomechanical effect of an implanted ACL graft by determining the tunnel position according to the ASR of the distal femur during flexion-extension motion. It was hypothesized that the ASR is a major factor affecting the biomechanical stability of ACLR surgery.

## 2. Materials and Methods

This study was conducted in compliance with the law about the Act on Dissection and Preservation of Corpses of the Republic of Korea (act number: 14885). All methods were performed in accordance with the relevant guidelines and regulations from the Catholic Institute for Applied Anatomy (Project identification number: R19-A027). The CT images used in this study were selected from the Catholic Digital Human Library, which was constructed by CT scans of the cadaver with the approval of the Institutional Review Board of College of Medicine, the Catholic University of Korea (No.: CUMC10U161). Written informed consent for the use of the cadaver and consent for the use of future research on the related materials were provided by all donors or authorized representatives.

### 2.1. Materials and Aspect Ratio Analysis

To analyze biomechanical characteristics according to the ASR of the knee joint, samples were selected from the Catholic Digital Human Library. The Catholic Digital Human Library is a collection of CT images from whole bodies of cadavers [17–19]. Only male samples were selected to exclude the effects of gender [15, 20], and 89 samples were selected for measurement. The mean age was 50.73 ± 9.99 years, and the mean height was 165.22 ± 6.30 cm. To generate the CT images, the slice thickness was set to 0.75–1.0 mm and the pixel value was set to 0.345–0.832 mm. The obtained images were reconstructed into 3D skeletal models using an image based on a 3D reconstruction program (Mimics, Ver. 20; Materialize, Belgium). Samples showed no congenital anomalies or pathological deformities around the knee joint. The maximum length parallel to the posterior condylar line was defined as the mediolateral (fML) width, whereas the maximum lengths of the lateral femoral condyles perpendicular to the posterior condylar line were defined as lateral anteroposterior (fAP) heights, respectively (Figure 1). The ASR at the distal femur was defined as the ratio of the height of the lateral condyles and the maximal mediolateral width (fML/fAP × 100, Figure 1). A small ASR indicates a circular knee shape, and a large ASR indicates a mediolateral long elliptical shape. After assessing the ASR range of the target models, we categorized the models into four stages through histogram analysis. The four representative models were selected for each group and used as analytical models.

### 2.2. Kinematical Tracking for Continuous Flexion/Extension of the Knee Joint

By capturing continuous movements of knee flexion/extension and inputting the trajectory data for knee movements to a computational analysis model, we aimed to implement a continuous knee movement that is different from the discrete knee movements reported in the existing studies. First, the shape of the cadaver femur and tibia was reconstructed with a 3D laser scanner (Faro Arm, Lake Mary, FL, USA) [6]. Four male cadaver knees were used. The mean age of the cadavers was 52.25 (44~60) years, and the mean height was 167.25 (163~171) cm. To accurately track knee flexion and extension motions, three markers were attached to major landmarks on each femur and tibia: the lateral collateral ligament attachment point, the medial collateral ligament attachment point, and the popliteal muscle-tendon complex. The kinematical data for each marker were captured at 15° intervals using motion capturing and image processing software (Geomagic 10, 3D System, Morrisville, USA). The 3-dimensional positions of each marker were fit to the shape of the femur and knee. Continuous knee movements were then extracted from discrete kinematical data measured at 15-degree intervals using the 5th-order spline interpolation technique to avoid undesired oscillation of the kinematic data during the dynamic simulation. During knee flexion and extension, the gap between the articular surfaces of the tibia and the femur was set at 2 mm [21, 22].

### 2.3. Three-Dimensional Multiflexible Body Dynamic Model and Femoral and Tibial Tunnel Placement

In this study, a reconstructed three-dimensional knee joint model based on CT images was implemented using Mimics 20.0 (Materialize, Leuven, Belgium), and a dynamic analysis model was generated that applied kinematical tracking data for the knee that was already secured in the cadaver. In addition, this study is aimed at gaining insight into the optimal surgical site by analyzing the stress applied to the implanted ACL graft considering ASR of the knee joint under continuous motion. Therefore, the dynamic analysis model implemented in this study was made into a multiflexible body dynamic (MFBD) model that reduced analysis time by setting the implanted ACL graft as a deformable flexible body (finite element model) capable of being analyzed for stress effects, and the implanted ACL graft was set as a rigid body. For this, a model capable of MFBD analysis was implemented using a commercial analysis program (RecurDyn V9R1, FunctionBay, Korea) [6].

**Figure 2:**
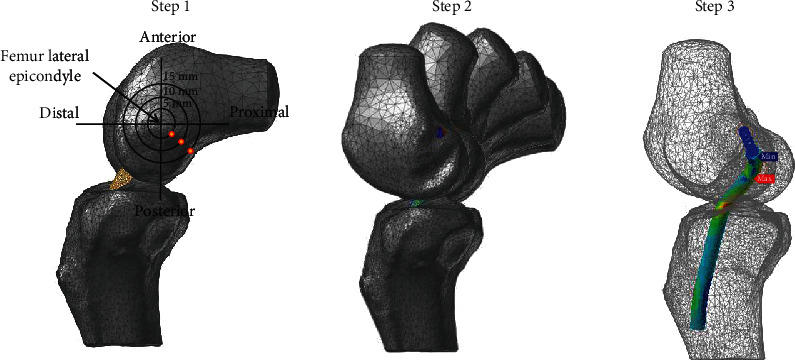
The process of computational analysis on ACLR for the 3D knee model showing recommended tunnel entry points during continuous motions; Step 1: the starting point for the three investigated femoral tunnels was the 45° posterior-proximal angle at 5 mm (A), 10 mm (B), and 15 mm (C) distances from the lateral epicondyle. Step 2: follow the shape of the knee using motion analysis data. Step 3: calculate stresses of the implant.

Next, in order to apply the outside-in surgical technique to the implemented 3D MFBD model, we drilled a surgical tunnel with 8 mm diameter suitable for the femur and tibia, respectively, by using the analysis program's editing tool. A tibial tunnel was created through the proximal cross-section using the anatomical axis measurement method on anthropometric measurements, and the femoral footprint was obtained from the sagittal plane using Bernard et al.'s quadrant method [23, 24]. The starting point for the three investigated femoral tunnels was the 45° posterior-proximal angle with distances of 5 mm (A), 10 mm (B), and 15 mm (C) from the lateral femoral epicondyle, as recommended by our previous analyses (Figure 2) [6].

### 2.4. Multiflexible Body Dynamic Analyses for Outside-In ACLR Surgeries

Among ACLR techniques, the outside-in method was adopted in this study. For this, both ends of the graft were attached to the MFBD model with the femoral and tibia tunnels. First, the implanted single-bundle ACL graft was modeled as a cylindrical flexible body with a diameter of 7.9 mm and a length of 100 mm, respectively [23, 25]. As mentioned earlier, the implanted ACL graft was designed as a flexible body (finite element model), and the number of elements was 5,594, the number of nodes was 6,520, and the element size was 1 mm. The material properties of the implanted ACL graft were set to 111 MPa Young's modulus and 0.46 Poisson ratio referred to existing studies [6, 26]. Next, the surface contact condition between the graft and the inside of each tunnel was applied to avoid penetration of each other. Since the implanted ACL graft was modeled as a flexible body and the tunnels of the femur and tibia as rigid bodies, the contact conditions were based on Hertz contact theory to avoid boundary overlap. The contact stiffness and contact damping were set to 10 N/mm and 0.0001, respectively, at which the boundaries between the flexible and rigid bodies did not overlap. The flexible body model was fixed at the beginning of the tibial tunnel and femoral tunnel using spring and bushing elements, respectively. A bushing force (stiffness 226 N/mm) was applied functionally to replace an interference screw at the starting point of the tibial tunnel [27]. To prepare for a case in which the graft was short, a button fixture (stiffness 1589.9 N/mm) with a 20 mm loop was connected to the implanted graft at the femoral tunnel starting point [6, 28]. This rigid body knee joint model with deformable ACL was based on our previous analyses and was validated through those studies [6].

**Figure 3:**
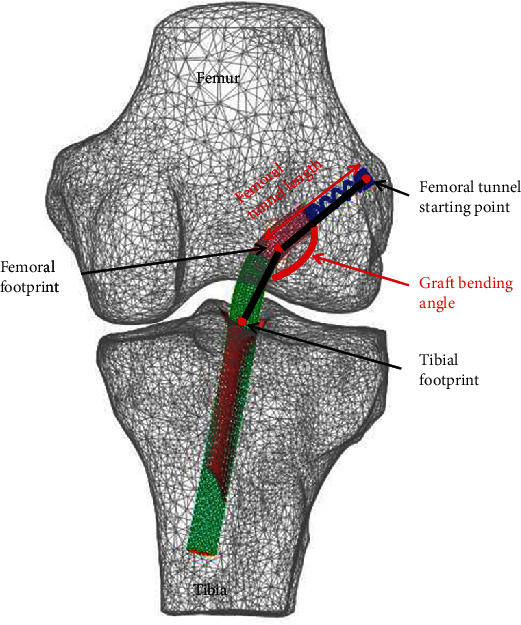
Description of the graft bending angle and femoral tunnel length.

For dynamic simulation, MFBD analyses by using simulation package (Recurdyn V9R1, FunctionBay, Korea) for 12 cases were performed by simulating four different ASR knee models (84%, 88%, 93%, and 97%) at three recommended tunnel entry points (posterior-proximal sites at 5, 10, and 15 mm distances from the lateral femoral epicondyle; Figure 2). Then, we compared the calculated von Mises stresses of the ACL graft, the femoral tunnel lengths, and the graft bending angles for each placement of the implanted graft in the knee models (Figure 3).

## 3. Results

### 3.1. Aspect Ratio of the Distal Femur

**Figure 4:**
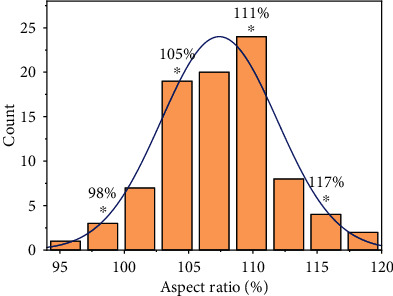
Histogram of knee ASRs (total 89 samples); simulation samples were selected at 98%, 105%, 111%, and 117%.

The average mediolateral (fML) width was 69.32 ± 3.50 mm. The average lateral anteroposterior (fAP) height was 64.63 ± 3.65 mm. The calculated ASR was from 95% to 119% (Figure 4). We selected ASRs at 98%, 105%, 111%, and 117% for biomechanical analysis because they represented quartile values across the distribution of knee samples.

### 3.2. Length of Femoral Tunnel

**Figure 5:**
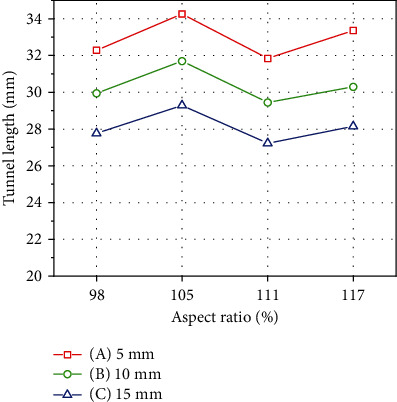
Calculated femoral tunnel length of patient-derived ACL graft at 5 mm, 10 mm, and 15 mm tunnel entry point distance from the lateral epicondyle. Femoral tunnel length decreased as the tunnel entry point distance from the lateral femoral epicondyle increased.

Regarding femoral tunnel lengths of the four different ASR knee models, the shortest femoral tunnel was 27.22 mm at 45° of the posterior-proximal direction at a distance of 15 mm (C) and an ASR of 111%, whereas the longest femoral tunnel was 34.25 mm at a distance of 5 mm (A) and an ASR of 105%. The average femoral tunnel lengths were 32.93 mm, 30.34 mm, and 28.11 mm when the distance was 5 mm (A), 10 mm (B), and 15 mm (C), respectively. Femoral tunnel length decreased as the distance increased at the 45° posterior-proximal direction from the lateral femoral epicondyle. The average femoral tunnel lengths were 29.99 mm, 31.74 mm, 29.50 mm, and 30.60 mm at ASRs of 98%, 105%, 111%, and 117%, respectively (Figure 5).

### 3.3. Bending Angle of the Implanted Graft

**Figure 6:**
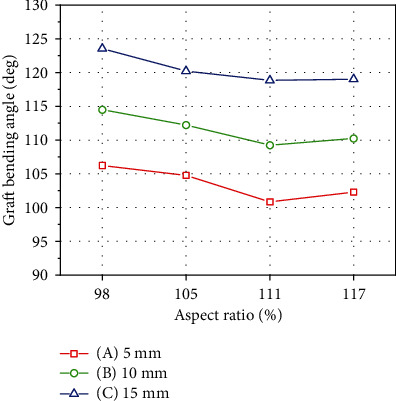
Calculated graft angles of ACL reconstruction at 5 mm, 10 mm, and 15 mm tunnel entry point distances from the lateral epicondyle. The graft bending angle increased as the tunnel entry point distance from the lateral femoral epicondyle increased.

Regarding the graft bending angles of the four different ASR knee models, the smallest graft bending angle was 100.85° with a distance of 5 mm (A) and an ASR of 111%, whereas the largest graft bending angle was 123.53° with a distance of 15 mm (C) and an ASR of 98%. The average graft bending angles were 103.52°, 111.55°, and 120.4° when the tunnel entry positions were 5 mm (A), 10 mm (B), and 15 mm (C), respectively. The graft bending angle increased as the distance increased from the lateral femoral epicondyle. The average graft bending angles at ASRs of 98%, 105%, 111%, and 117% were 114.75°, 112.40°, 109.64°, and 110.51°, respectively (Figure 6).

### 3.4. von Mises Stress of Implanted Grafts during Continuous Knee Motion

**Figure 7:**
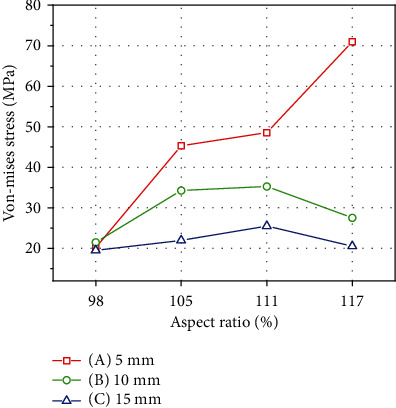
Maximum von Mises stress in grafts according to knee ASR. Stress decreased with increasing distance at ASRs of 105%, 111%, and 117%. The stress at an ASR of 98% changed very little with increasing distance from the lateral epicondyle.

**Figure 8:**
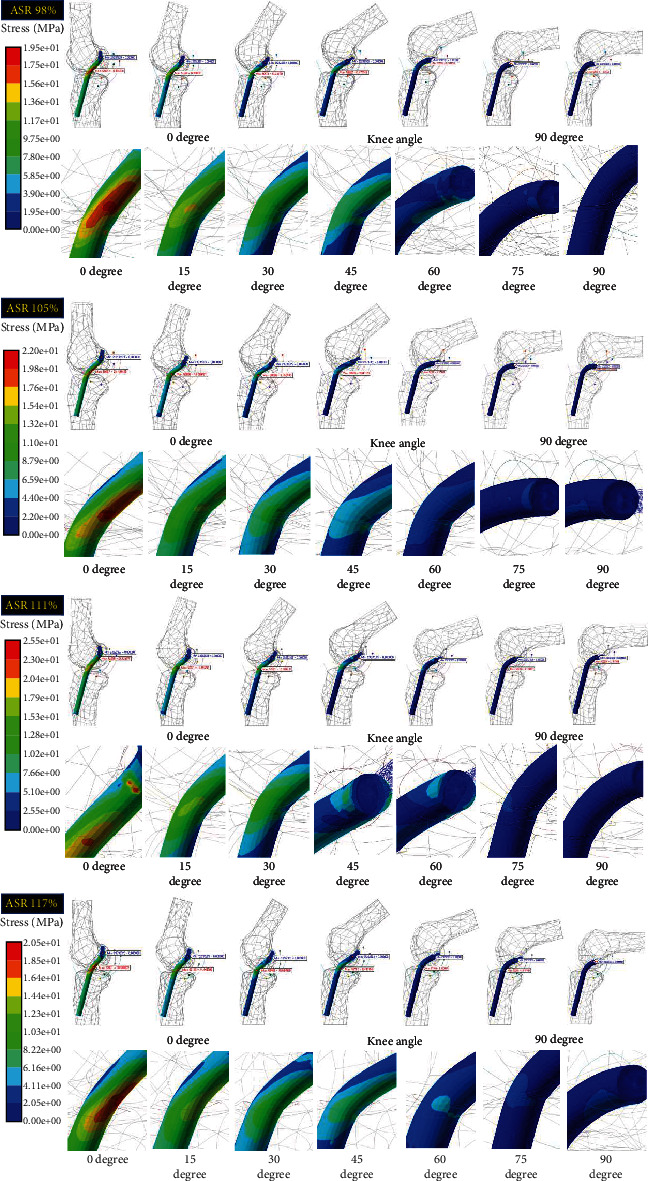
Calculated von Mises stress contour of implanted ACL grafts at seven major angles during continuous knee flexion/extension movement as a result of MFBD analyses at the various ASRs: Max. stress (red) and Min. stress (blue).

Regarding the von Mises stress of the implanted grafts in the four different ASR knee models during continuous knee movement, the lowest von Mises stress was 19.4 MPa at the 45° posterior-proximal angle at a distance of 15 mm (C) and an ASR of 98%, whereas the highest von Mises stress was 71 MPa at the 45° posterior-proximal angle with a distance of 5 mm (A) and an ASR of 117% (Figure 7). The von Mises stress of the implanted graft decreased with increasing distance at ASRs of 105%, 111%, and 117%, but the von Mises stress of the implanted graft at an ASR of 98% changed very little as the distance from the lateral epicondyle increased. The von Mises stress of the implanted graft during continuous knee movement also increased as the knee joint angle decreased. The highest stress value was observed when the knee joint angle was between 0° and 30° (Figure 8).

## 4. Discussion

The main cause of ACLR surgery failure is incorrect tunnel position [29, 30]. This mispositioning causes damage to knee structures (cartilage, ligaments, meniscus, etc.) and could lead to arthritis [31]. The main purpose of ACL surgery is stabilization to prevent early knee wear. If the femoral tunnel is placed anteriorly, it causes tension in the graft, limiting flexion and rotational stability [32]. To reduce failure and improve stability during ACLR surgery, surgical methods considering anatomy, such as the outside-in technique, have been introduced [7, 33]. In this study, tunnel position was selected based on the ASR of the knee through the outside-in technique during ACL surgery.

For successful ACL reconstruction, many researchers have focused on the optimal tunnel position with tunnelling technique through biomechanical simulations [6, 25, 34, 35]. However, anatomical characteristics of the knee were not considered in these studies. Computational studies of the optimal tunnel position mentioned above have been carried out without any consideration of these anatomical characteristics. Therefore, in this study, we investigated the biomechanical effect of the implanted graft on the optimal tunnel position for four representative ASRs.

According to Beckers et al. [36], men had wider knees (range 108~151%) than women (range 106~146%), and compared to Caucasian knees (range 116~126%), Arabian (range 106~149%) and Indian (range 118~147) knees were wider, while East Asian knees were narrower (range 100~118). This study on Korean men showed knee ASRs of 95~119%, similar to the results of a previous study on Asians [37, 38]. The femoral tunnel length for ACL graft was not affected by the ASR of the distal femur. The femoral tunnel length at 111% ASR showed a minor decrement compared with 117% ASR. The tunnel length at 105% ASR increased and then decreased at 98% ASR. Finally, the tunnel length at 117% ASR was similar to the length at 98% ASR (Figure 6). The changes in tunnel length among the various ASRs were about 1 mm, and the shortest tunnel length was 27.22 mm. Existing studies found that the minimum required tunnel length was 25 mm for successful reconstruction surgery [35, 39]. Thus, the ASR of the distal femur might not be an essential factor for determining adequate tunnel length.

The calculated value of the graft bending angle showed an increase when the entry point was far from the lateral epicondyle. The results at 111% and 117% ASRs were similar or were slightly decreased compared with the increase at 98% and 105% ASRs (Figure 7). The graft bending angle had a larger value as ASR decreased at every entry point. Thus, surgeons should pay attention to the graft bending angle when confronted with a knee with a small ASR. A small ASR causes a large value for the graft bending angle, and the graft bending angle affects early graft healing and tunnel widening [40–42].

Based on the review of previous computational studies, accurate comparisons were limited due to the lack of information on the ASRs of the analysis models. According to the study of finite element analysis by Song et al. [43], the von Mises stress distribution of a single bundle was 16.5 to 24 MPa. In addition, Westermann et al. performed FEA studies about the various diameters (5 to 9 mm) of ACL grafts with a knee angle of 20 degrees [44]. Their results showed a von Mises stress distribution of 15 to 22 MPa. Lastly, in the study of Wan et al. [45], a three-dimensional FEA analysis of ACL reconstruction was performed using three grafts at a knee angle of 0 degrees. As a result, the stress distribution was 20 to 52 MPa. Our study showed the highest stress of the implanted ACL graft at a knee angle of 0 to 15 degrees for the four ASR models. In addition, our study showed calculated stress values (19.49 to 71 MPa) in the range of those from previous studies because various tunnel positions and ASRs were considered in the analysis.

The maximum stress in the graft, when the ASR was 117%, had a high-stress value with the tunnel entry point near the lateral epicondyle. The maximum stress value at a 5 mm distance from the lateral epicondyle was 3.5 times higher than the 15 mm entry position, and in the cases at 105% and 111% ASR, the stress was 1.9 times higher at a 5 mm distance compared with a 15 mm distance. Otherwise, in cases at 98% ASR, a low-stress value was achieved at all entry points. When reconstructing the ACL with a large ASR, creating a femoral tunnel entry point close to the lateral epicondyle can increase stress on the graft.

The differences in results were small in the ASR 105-111% range, which accounted for most of the sample. However, the difference of the bending angle was observed with a large ASR of 117%, which is close to an ellipse, and the maximum stress increased at small ASR (98%), which is close to a circular shape. Considering the number of samples in this study (89 total), an 117% ASR was found in 10.11% (9/89) of the total sample, and a 98% ASR was found in 8.89% (8/89). Therefore, about 20% of the samples had a small or large ASR; at least in these samples, the difference in ASR should be considered during ACLR surgery.

This study had some limitations. First, we had to use cadavers to analyze knee kinematics in the flexion-extension process. Passive movement was performed without consideration of body weight. In addition, major ligaments and soft tissues such as the medial/lateral collateral ligament, posterior cruciate ligament, and anterolateral ligament were not considered. However, the main role of these soft tissues is involvement in passive movement. Second, the results were not validated by biomechanical testing after computational analysis of graft tensioning according to ASR and tunnel entry position. Such analysis was not possible here. A biomechanical test with three recommended tunnel entry points with one ASR requires at least three cadavers with the same ASR. In addition, it is difficult to produce three tunnels in a femur with the same ASR. However, this study confirmed that kinematic data obtained from limited conditions and various ASRs of the knee can affect movement, and these factors can affect ACL graft in small or large ASRs.

## 5. Conclusions

This study is aimed at confirming through computational analysis whether ASR, which was not examined in previous studies, should be considered for current surgical procedures. Our computations showed that there is no relationship between ASR and femoral tunnel length for ACLR surgery. A smaller ASR causes a higher graft bending angle, and a larger ASR causes greater stress to the graft. As a result, the ASR of the distal femur is a factor that should be considered in ACLR surgical plans. Small or large ASRs should be considered during ACLR surgery. We also believe that clinical studies on possible graft stress outcomes will be necessary.

## Data Availability

The datasets used and/or analyzed during the current study are available from the corresponding author on reasonable request.

## References

[1] Lien-Iversen T., Morgan D. B., Jensen C., Risberg M. A., Engebretsen L., Viberg B. (2020). Does surgery reduce knee osteoarthritis, meniscal injury and subsequent complications compared with non-surgery after ACL rupture with at least 10 years follow-up? A systematic review and meta-analysis. *British Journal of Sports Medicine*.

[2] Kvist J., Filbay S., Andersson C., Ardern C. L., Gauffin H. (2020). Radiographic and symptomatic knee osteoarthritis 32 to 37 years after acute anterior cruciate ligament rupture. *The American Journal of Sports Medicine*.

[3] Ardern C. L., Webster K. E., Taylor N. F., Feller J. A. (2010). Hamstring strength recovery after hamstring tendon harvest for anterior cruciate ligament reconstruction: a comparison between graft types. *Arthroscopy*.

[4] Ardern C. L., Webster K. E., Taylor N. F., Feller J. A. (2011). Return to the preinjury level of competitive sport after anterior cruciate ligament reconstruction surgery: two-thirds of patients have not returned by 12 months after surgery. *The American Journal of Sports Medicine*.

[5] Jaecker V., Zapf T., Naendrup J. H. (2017). High non-anatomic tunnel position rates in ACL reconstruction failure using both transtibial and anteromedial tunnel drilling techniques. *Archives of Orthopaedic and Trauma Surgery*.

[6] Kang K., Bae T. S. (2017). Effect of femoral tunnel positions on graft stress in outside-in ACL reconstruction surgery during continuous knee motion: a simulation study. *The International Journal of Medical Robotics and Computer Assisted Surgery*.

[7] Ji G., Han A., Hao X., Li N., Xu R., Wang F. (2018). Better rotational control but similar outcomes with the outside-in versus the transtibial drilling technique for anterior cruciate ligament reconstruction: a systematic review of comparative trials. *Archives of Orthopaedic and Trauma Surgery*.

[8] Iriuchishima T., Goto B. (2020). Systematic review of surgical technique and tunnel target points and placement in anatomical single-bundle ACL reconstruction. *The Journal of Knee Surgery*.

[9] Lee S. R., Jang H. W., Lee D. W., Nam S. W., Ha J. K., Kim J. G. (2013). Evaluation of femoral tunnel positioning using 3-dimensional computed tomography and radiographs after single bundle anterior cruciate ligament reconstruction with modified transtibial technique. *Clinics in Orthopedic Surgery*.

[10] Shino K., Suzuki T., Iwahashi T. (2010). The resident's ridge as an arthroscopic landmark for anatomical femoral tunnel drilling in ACL reconstruction. *Knee Surgery, Sports Traumatology, Arthroscopy*.

[11] Zauleck M. K., Gabriel S., Fischmeister M. F., Hirtler L. (2014). Origin of the anterior cruciate ligament and the surrounding osseous landmarks of the femur. *Clinical Anatomy*.

[12] Nam-Ki K., Jong-Min K. (2015). The three techniques for femoral tunnel placement in anterior cruciate ligament reconstruction: transtibial, anteromedial portal, and outside-in techniques. *Arthroscopy and Orthopedic Sports Medicine.*.

[13] Cho H. J., Kwak D. S., Kim I. B. (2015). Morphometric evaluation of Korean femurs by geometric computation: comparisons of the sex and the population. *BioMed Research International*.

[14] Verma M., Joshi S., Tuli A., Raheja S., Jain P., Srivastava P. (2017). Morphometry of proximal femur in Indian population. *Journal of Clinical and Diagnostic Research*.

[15] Hitt K., Shurman J. R., Greene K. (2003). Anthropometric measurements of the human knee: correlation to the sizing of current knee arthroplasty systems. *Journal of Bone and Joint Surgery*.

[16] Kwak D. S., Surendran S., Pengatteeri Y. H. (2007). Morphometry of the proximal tibia to design the tibial component of total knee arthroplasty for the Korean population. *The Knee*.

[17] Kim S., Kwak D. S., Kim I. B. (2017). Morphometric analysis and classification of the cross-sectional shape of the C2 lamina. *Biomed Research International*.

[18] Park S. A., Kwak D. S., Cho H. J. (2019). Technical variation of trans-articular sacroiliac joint (SIJ) fusion using three screws considering the effects of sacral dysplasia in patients with non-traumatic SIJ pain. *Bmc Musculoskeletal Disorders*.

[19] Park S. A., Kwak D. S., You S. L. (2015). Entry zone of iliac screw fixation to maintain proper entry width and screw length. *European Spine Journal.*.

[20] Csintalan R. P., Schulz M. M., Woo J., McMahon P. J., Lee T. Q. (2002). Gender differences in patellofemoral joint biomechanics. *Clinical Orthopaedics and Related Research*.

[21] Faisal A., Ng S. C., Goh S. L., Lai K. W. (2018). Knee cartilage segmentation and thickness computation from ultrasound images. *Medical & Biological Engineering & Computing*.

[22] Schmitz R. J., Harrison D., Wang H. M., Shultz S. J. (2017). Sagittal-plane knee moment during gait and knee cartilage thickness. *Journal of Athletic Training*.

[23] Bernard M., Hertel P., Hornung H., Cierpinski T. (1997). Femoral insertion of the ACL. Radiographic quadrant method. *The American journal of knee surgery*.

[24] Ko Y. W., Rhee S. J., Kim I. W., Yoo J. D. (2015). The correlation of tunnel position, orientation and tunnel enlargement in outside-in single-bundle anterior cruciate ligament reconstruction. *Knee Surgery & Related Research*.

[25] Bae T. S., Cho B. C. (2020). Biomechanical effect of tunnel positions and pre-tension forces on implanted graft stress and strain during outside-in ACL reconstruction surgery: a simulation study. *International Journal of Precision Engineering and Manufacturing.*.

[26] Noyes F. R., Grood E. S. (1976). The strength of the anterior cruciate ligament in humans and Rhesus monkeys. *The Journal of Bone and Joint Surgery. American Volume*.

[27] Bloemker K. H., Guess T. M., Maletsky L., Dodd K. (2012). Computational knee ligament modeling using experimentally determined zero-load lengths. *The Open Biomedical Engineering Journal*.

[28] Jin C., Paluvadi S. V., Lee S., Yoo S., Song E. K., Seon J. K. (2018). Biomechanical comparisons of current suspensory fixation devices for anterior cruciate ligament reconstruction. *International Orthopaedics*.

[29] Khalfayan E. E., Sharkey P. F., Alexander A. H., Bruckner J. D., Bynum E. B. (1996). The relationship between tunnel placement and clinical results after anterior cruciate ligament reconstruction. *The American Journal of Sports Medicine*.

[30] Pinczewski L. A., Salmon L. J., Jackson W. F. M., von Bormann R. B. P., Haslam P. G., Tashiro S. (2008). Radiological landmarks for placement of the tunnels in single-bundle reconstruction of the anterior cruciate ligament. *Journal of Bone and Joint Surgery. British Volume (London)*.

[31] Samitier G., Marcano A. I., Alentorn-Geli E., Cugat R., Farmer K. W., Moser M. W. (2015). Failure of anterior cruciate ligament reconstruction. *The Archives of Bone and Joint Surgery*.

[32] Rayan F., Nanjayan S. K., Quah C., Ramoutar D., Konan S., Haddad F. S. (2015). Review of evolution of tunnel position in anterior cruciate ligament reconstruction. *World Journal of Orthopedics*.

[33] Robin B. N., Jani S. S., Marvil S. C., Reid J. B., Schillhammer C. K., Lubowitz J. H. (2015). Advantages and Disadvantages of Transtibial, Anteromedial Portal, and Outside- In Femoral Tunnel Drilling in Single-Bundle Anterior Cruciate Ligament Reconstruction: A Systematic Review. *Arthroscopy*.

[34] Forsythe B., Kopf S., Wong A. K. (2010). The location of femoral and tibial tunnels in anatomic double-bundle anterior cruciate ligament reconstruction analyzed by three-dimensional computed tomography models. *The Journal of Bone and Joint Surgery. American Volume*.

[35] Guglielmetti L. G. B., Shimba L. G., do Santos L. C. (2017). The influence of femoral tunnel length on graft rupture after anterior cruciate ligament reconstruction. *Journal of Orthopaedics and Traumatology*.

[36] Beckers L., Müller J. H., Daxhelet J., Saffarini M., Aït-Si-Selmi T., Bonnin M. P. (2021). Sexual dimorphism and racial diversity render bone-implant mismatch inevitable after off-the-shelf total knee arthroplasty: a systematic review and meta-analysis. *Knee Surgery, Sports Traumatology, Arthroscopy*.

[37] Nishikawa M., Owaki H., Kaneshiro S., Fuji T. (2014). Preoperative morphometric differences in the distal femur are based on skeletal size in Japanese patients undergoing total knee arthroplasty. *Knee Surgery, Sports Traumatology, Arthroscopy*.

[38] Reddy A. V. G., Sankineani S. R., Agrawal R., Thayi C. (2020). Comparative study of existing knee prosthesis with anthropometry of Indian patients and other races, a computer tomography 3D reconstruction-based study. *Journal of Clinical Orthopaedics and Trauma*.

[39] Bedi A., Raphael B., Maderazo A., Pavlov H., Williams R. J. (2010). Transtibial versus anteromedial portal drilling for anterior cruciate ligament reconstruction: a cadaveric study of femoral tunnel length and obliquity. *Arthroscopy*.

[40] Chen L., Wu Y., Lin G. (2018). Graft bending angle affects allograft tendon maturity early after anterior cruciate ligament reconstruction. *Knee Surgery, Sports Traumatology, Arthroscopy*.

[41] Tashiro Y., Gale T., Sundaram V. (2017). The graft bending angle can affect early graft healing after anterior cruciate ligament reconstruction: in vivo analysis with 2 years' follow-up. *The American Journal of Sports Medicine*.

[42] Tashiro Y., Sundaram V., Thorhauer E. (2017). In Vivo Analysis of Dynamic Graft Bending Angle in Anterior Cruciate Ligament- Reconstructed Knees During Downward Running and Level Walking: Comparison of Flexible and Rigid Drills for Transportal Technique. *Arthroscopy*.

[43] Song Y., Debski R. E., Musahl V., Thomas M., Woo S. L. (2004). A three-dimensional finite element model of the human anterior cruciate ligament: a computational analysis with experimental validation. *Journal of Biomechanics*.

[44] Westermann R. W., Wolf B. R., Elkins J. M. (2013). Effect of ACL reconstruction graft size on simulated Lachman testing: a finite element analysis. *Iowa Orthopedic Journal*.

[45] Wan C., Hao Z., Wen S. The finite element analysis of three grafts in the anterior cruciate ligament reconstruction.

